# Effect of acute exercise intensity on cognitive inhibition and well-being: Role of lactate and BDNF polymorphism in the dose-response relationship

**DOI:** 10.3389/fpsyg.2022.1057475

**Published:** 2022-12-09

**Authors:** Juan Arturo Ballester-Ferrer, Beatriz Bonete-López, Alba Roldan, Eduardo Cervelló, Diego Pastor

**Affiliations:** ^1^Sports Research Centre, Department of Sport Sciences, Miguel Hernández University of Elche, Elche, Spain; ^2^Department of Psychology, Miguel Hernández University of Elche, Elche, Spain

**Keywords:** cognitive inhibition, exercise, wellbeing, lactate, BDNF

## Abstract

**Introduction:**

There is evidence in the literature that acute exercise can modify cognitive function after the effort. However, there is still some controversy concerning the most effective exercise modality to improve cognitive function in acute interventions. Regarding these different exercise modalities, the dose–response relationship between exercise intensity and cognitive response is one of the most challenging questions in exercise and cognition research.

**Methods:**

In this study, we tested the impact of moderate-intensity (MICT), high-intensity (HIIT) exercise sessions, or control situation (CTRL) on cognitive inhibition (measured with the Stroop Test). Thirty-six young college students participated in this study, where a within-subject repeated measure design was used.

**Results:**

ANOVA 2×3 demonstrated that HIIT improved the acute cognitive response to a higher degree when compared to MICT or CTRL (*p* < 0.05). The cognitive improvements correlated with lactate release, providing a plausible molecular explanation for the cognitive enhancement (*r* < −0.2 and *p* < 0.05 for all the Stroop conditions). Moreover, a positive trend in wellbeing was observed after both exercise protocols (HIIT and MICT) but not in the CTRL situation. Genetic BDNF single nucleotide polymorphism did not influence any interactions (*p* < 0.05).

**Discussion:**

In this sense, our results suggest that exercise intensity could be a key factor in improved cognitive function following exercise in young college students, with no additional impact of BDNF polymorphism. Moreover, our results also provide evidence that exercise could be a useful tool in improving psychological wellbeing.

## Introduction

Learning is a higher-order process emerging from the Central Nervous System and shaped through the interaction of diverse cognitive domains; among them, executive function (EF) plays a central role (Diamond, 2013). EF is a broad term that comprises a set of neurocognitive skills necessary for optimal daily functioning, academic success, and employability (Diamond, 2013; Titz and Karbach, 2014). These skills include inhibitory control (or simply inhibition), working memory, and cognitive flexibility (also termed mental set shifting; Diamond, 2013). Given the importance of the said skills across the lifespan, strategies for their optimization have been extensively explored in the literature. Physical exercise has emerged as one of the most promising tools to benefit all aspects of executive function (Chang et al., 2012; Stillman et al., 2016; Oberste et al., 2019; Pontifex et al., 2019; Stillman et al., 2020). Of all of them, Inhibitory control is the most studied domain out of the three core EFs in its relationship to the effects of acute physical exercise (Pontifex et al., 2019). Inhibitory control refers to the capacity of an individual to override strong impulses and focus attention on relevant stimuli, thus, ensuring goal-oriented behaviors (Nigg, 2000; Friedman and Miyake, 2004). Although a large body of research has focused on the benefits of physical exercise on cognition in general and inhibition in particular, evidence examining the underlying mechanisms is still lacking (Stillman et al., 2016, 2020).

The dose–response relationship of both acute and chronic exercise has gained attention as an easily modifiable moderator variable in the exercise-cognition literature (Chang et al., 2012, 2015; Herold et al., 2019; Oberste et al., 2019; Ludyga et al., 2020). Evidence suggests that as long as a rest interval is introduced after a single bout of aerobic exercise, higher intensities should result in larger improvements in cognition (Chang et al., 2012; Oberste et al., 2019). Along the same lines, exercise intensity was shown to modulate the exercise response-inhibition relationship, with high-intensity interval training (HIIT) producing superior results, followed by vigorous continuous exercise. Conversely, only a small benefit was reported for moderate continuous training (MICT) compared to a no-exercise control condition (Oberste et al., 2019). In an attempt to elucidate the underlying mechanisms of the findings above and expand our knowledge of the exercise-cognition relationship, the vast majority of the studies have focused on molecular and cellular interactions and structural and functional cerebral changes (Stillman et al., 2016). In this line of evidence, high-intensity exercise was shown to prompt the release of neurotrophic factors, such as Brain-Derived Neurotrophic Factor (BDNF; Saucedo Marquez et al., 2015). The implication is that BDNF mediates cognitive enhancement following acute exercise, and this relationship, in turn, could be related to lactate production (Ferris et al., 2007). In fact, recent studies have demonstrated that lactate released from the exercising muscle during a high-intensity bout could cross the brain–blood barrier (El Hayek et al., 2019; Nicola and Okun, 2021) and induce BDNF expression in the brain (Müller et al., 2020). Lactate, thus, has been proposed as a key molecule in brain health and the observed cognitive enhancement following physical exercise (El Hayek et al., 2019; Hashimoto et al., 2021). However, some studies have suggested the inverted U-shaped relationship between exercise and cognition. According to this theory, moderate-intensity exercise protocols granted the most cognitive gains rather than those that incorporated exercise of lighter or higher intensities (Pontifex et al., 2019).

It has been argued that molecular and cellular modifications alone cannot explain the exercise-cognition relationship, and other socioemotional and behavioral aspects could be implicated, but the literature concerning social and behavioral aspects is scarce (Stillman et al., 2016). In this regard, exercise could improve psychological wellbeing (Cervelló et al., 2014). At the same time, psychological wellbeing is viewed as a modulator of the learning process, with previous research highlighting its central role in academic achievement in young students (Garcia et al., 2015; Yu et al., 2017). Moreover, some studies have hypothesized that low cognitive performance could affect psychological wellbeing, leading to mental health issues in the present or in the future (Leahy et al., 2020), and although there is still no agreement on what defines psychological wellbeing (Korhonen et al., 2014), a few perspectives on its study have come to light (Ryan et al., 2013). The hedonic perspective interprets wellbeing as the presence of positive affect and the absence of negative affect. In contrast, the eudemonic perspective associates wellbeing with an individual’s ability to experience and exercise their human potential to achieve optimal psychological functioning (Ryan et al., 2013). Thus, understanding the benefits of physical exercise on cognition and psychological wellbeing, as well as the underlying pathways, could be of great importance for the integral health of the population.

In addition to modifiable factors mentioned above that were implicated in the cognitive response to exercise, other non-modifiable aspects, such as genetic profile, could potentially affect the individual response (Canivet et al., 2015). In this regard, levels of BDNF, a protein that mediates the effects of exercise on the brain and, by extension, on cognition (Cotman et al., 2007), have been reported to increase following exercise bout in an intensity-dependent manner (Ferris et al., 2007). Nonetheless, there is evidence that some individuals may not see the exercise-induced rise in BDNF, given precisely their genetic profile. In this respect, the BDNF gene regulates the release of BDNF; the single nucleotide polymorphism (SNP) of the gene, called SNP rs6262 or BDNF Val66Met gene polymorphism, substitutes a valine (Val) for methionine (Met) at codon 66. Those with methionine (Met/Met) polymorphism respond with lower BDNF levels to exercise (Egan et al., 2003); this observation could explain the reduced effects of exercise on cognition in these individuals (Canivet et al., 2015). Nonetheless, contrasting hypotheses exist on the relationship between cognitive response and BDNF polymorphism (de Las Heras et al., 2022). Some studies have reported a better cognitive performance in the Val carriers following exercise, compared to the Met carriers, given that the Val polymorphism is associated with higher activity-dependent BDNF release (Mang et al., 2013). On the other hand, some authors have proposed that activity-dependent BDNF secretion is less efficient in the presence of the methionine SNP, and thus, engagement in physical exercise, which should produce a rise in BDNF levels, would ultimately lead to an improved cognitive response in the Met carriers, rather than those with the valine SNP (Moreau et al., 2017). In Spain, the Val genotype is the most common (65.6–53.3%), followed by the Val/Met polymorphism (33.3–30.7%), and finally, the Met/Met genotype (13.3–3.7%; Combarros et al., 2004; Sánchez-Romero et al., 2009). Given these divergent theories, it appears necessary to advance our understanding of the implications of BDNF SNPs for an individual cognitive response.

For all the above reasons, the study’s main objective was to explore the impact of exercise intensity on inhibitory control and psychological wellbeing. Moreover, we have also hypothesized that lactate released during exercise bout would be associated with Post-exercise cognitive enhancement, and genetic profile, such as BDNF polymorphism, would mediate cognitive response following exercise. In this regard, we have hypothesized that the participants with at least one Met allele would experience reduced exercise effects on their cognitive function.

## Materials and methods

### Subjects

Thirty-six university students (19 female subjects) were enrolled in the study. The sample size was estimated using G-Power for a ANOVA RM 2×3 repeated measures (1 group, 6 measures), where alpha was 0.05, power was 0.8, and the estimated *n*_p_^2^ > 0.05 was assumed. Given the variables of interest, we estimated that the required sample size should include 33 participants. Thirty-six participants were recruited to account for possible dropouts. In a *post hoc* analysis, the ANOVA RM showed a *η*_p_^2^ of 0.37 (congruent condition), 0.13 (neutral condition), and 0.19 (incongruent condition), meaning our study’s power was >0.99 in all conditions. Subjects were also asked to fill in the “Physical Activity Readiness Questionnaire” (PAR-Q) to ensure that neither of them had any contraindications to participating in an exercise protocol. Participants were asked to sign the informed written consent form prior to their inclusion in the study. The study protocol was guided in the most recent (7th) Declaration of Helsinki and was approved by the University Ethics Committee (UMH.CID.DPC.02.17). The characteristics of the participants are summarized in Table 1.

**Table 1:** Sample description.

Variable	Characteristics
Age (years)	21.97 ± 2.56
Height (m)	1.69 ± 0.09
Weight (kg)	70.07 ± 11.26
BMI (kg.m^2^)	24.39 ± 2.72
Fat mass (%)	22.49 ± 7.13
FC max (bpm)	196.56 ± 8.03
MAS (km/h)	14.95 ± 1.41

### Measurements

#### Cognitive function

##### Stroop test

The Stroop test (Stroop, 1935) is one of the most widely used neuropsychological instruments to assess several core cognitive processes, including executive function, selective attention, and inhibitory control (Golden, 1994; Chang et al., 2015, 2019). In our study, a digital version of the test was applied (Stroop test UMH-MEMTRAIN by Pastor et al., 2018), which allowed the assessment of reaction time and accuracy, following the original protocol described by Golden (1994). Eight-inch tablets (Lenovo TB3-850F) were provided to the participants to complete the test. The test comprises three stages of increasing difficulty. The first one represents a congruent condition, the second one represents the neutral condition, and the third trial is “incongruent” for the reasons detailed below. During each trial, one of four stimuli (the words “red,” “blue,” “yellow,” or “green” in its Spanish translation or “XXXX”) is displayed in the center of a screen on a white background. Meanwhile, the bottom of the screen features 4 buttons with the 4 possible answers in black ink (Spanish names for red, blue, yellow, or green) for each of the test conditions. In the first congruent condition, the color names (Spanish names for red, blue, yellow, or green) appear on the screen in black ink, and the subjects must match the word to the color it denotes from the bottom four options. Meanwhile, in the second neutral condition, rather than the color name, “XXXX” is displayed in any color ink mentioned earlier. During the third incongruent condition, yet again the color name appears on the screen, but the color name does not correspond to the color ink of the word (i.e., the word “red” appears in green ink). In this case, the participants should respond to the color of the ink, rather than the the meaning of the word. Participants were instructed to select the correct option “as quickly and accurately as possible.” Each condition lasts 45 s, where the number of correct and incorrect answers is recorded. The test pauses for 25 s before starting the next condition. The dependent measurements are divided into three levels: (1) Congruent condition, (2) Neutral condition, and (3) Incongruent condition. Prior to the experimental condition, five familiarization trials of the Stroop task were carried out to reduce the learning effect. For the main analysis, reaction times and response accuracy were included. In our study, before starting the experimental protocol, we found the intraclass correlation coefficient (ICC) of reaction time in each of the conditions of the Stroop test (congruent: ICC = 0.76; neutral: ICC = 0.76; incongruent: ICC = 0.79) was high (>0.70; Rodríguez Barreto et al., 2016).

##### Stroop test check

Interactions between all three test conditions and three experimental protocols were analyzed to ensure adequate manipulation of the Stroop test. When the test is administered correctly, the incongruent condition should be perceived as the most complex compared to the two preceding conditions, independent of the external factors surrounding the test’s administration. As a consequence, ANOVA should reflect longer reaction times and worse precision rates in the incongruent condition before and after any experimental protocol. The drop-off in precision in the incongruent condition should also be similar between treatments.

The Pre-and Post-measurements of the Stroop test, for both reaction times and precision, were analyzed using ANOVA RM 3 × 3 (Congruent, Neutral, Incongruent × HIIT, MICT, CTRL).

For the reaction times, a main effect of the Stroop condition was demonstrated both in the Pre [*F*(1.43, 48.71) = 207.102, *p* < 0.001, *η*_p_^2^ = 0.86] and in the Post [*F*(1.44, 50.27) = 169.28, *p* < 0.001, *η*_p_^2^ = 0.83]. The same main effect (Stroop condition) was true for precision in both, the Pre [*F*(2, 66) = 10.98, *p* < 0.001, *η*_p_^2^ = 0.25] and the Post [*F*(1.64, 54.37) = 6.91, *p* < 0.01, *η*_p_^2^ = 0.17].

Moreover, the main effect of Treatment could not be demonstrated at either time point for precision rates (*p* > 0.05). For reaction times, on the other hand, although no effect of the Treatment was observed in the Pre (*p* > 0.05), it was found in the Post (*p* < 0.05).

Consequently, the incongruent condition resulted in the longest reaction times and the highest rate of precision errors when contrasted with the two remaining conditions (congruent and neutral). This finding agrees with those previously reported and is associated with the so-called Stroop effect (Mac Leod, 1991; Milham et al., 2002; Chang et al., 2019). It is worth highlighting that the precision ratios were similar between experimental protocols (HIIT, MICT, and CTRL). This latter observation, combined with the differences between Stroop conditions, indicates satisfactory manipulation of the Stroop test from our side.

#### Psychological wellbeing

##### Subjective vitality

The Subjective Vitality Questionnaire (Ryan and Frederick, 1997) was used to measure the perception of vitality before and after each experimental condition, adapted to Spanish by Molina-García et al. (2007). This questionnaire is considered a measure of psychological wellbeing (Ryan et al., 2013). The scale allows participants to express present-day feelings through seven items (e.g., I feel alive and vital). Responses are rated on an eight-point Likert-type scale ranging from 0 (not completely true) to 7 (very true). Cronbach’s alpha in the different experimental situations was comprised between 0.76 and 0.92.

##### Affective state

Before and after each experimental situation, participants were asked to complete the Positive and Negative Affect Schedule (Mackinnon et al., 1999), to assess their positive and negative emotional experiences. This questionnaire is considered a hedonic measure of wellbeing. The scale is made up of nine adjectives grouped into two factors. The subjects chose those adjectives, which allowed them to respond to the following sentence: “Indicate how you feel at this moment….” Four of the items are associated with Positive Affect (“cheerful, happy, contented, amused”) and five with Negative Affect (“depressed, worried, frustrated, angry, unhappy”). The instrument uses an eight-point Likert scale, ranging from 1 “not at all” to 8 “extremely.” Cronbach’s alpha ranged between 0.74 and 0.96 for the two factors in the present study.

#### Ramp incremental test

A graded exercise test on a treadmill was performed to establish Maximum Aerobic Speed (MAS); the latter was then used to calculate the relative intensities (i.e., speed) of both experimental conditions. The protocol included a 3-min warm-up at 5 km/h, followed by 1 km/h increments per minute until volitional exhaustion. A treadmill incline was set at 1% for both the warm-up and incremental stages. Heart rate (HR) was monitored throughout the test through H7 chest straps (Polar Electro Oy, Kempele, Finland) and allowed to register the maximum HR achieved by the end of the test. Participants were asked not to engage in any strenuous exercise 24 h prior to the test.

#### Intensity parameters

To determine blood lactate concentrations, samples were obtained from the participants’ earlobes 3 and 15 min after each of the experimental conditions. A portable lactate analyzer (Lactate Scout, SensLab GmbH, Germany) was used to employ the said evaluation.

HR was monitored during HIIT and MICT by means of H7 chest straps linked to the Polar Beat app (Polar Electro Oy, Kempele, Finland). The HR register was also utilized to ensure the participants adhered to the relative intensity corresponding to each experimental condition.

#### Genetic analysis

Saliva samples were collected with OrageneTM DNA Saliva Collection Kit (DNA Genotek S.L.). DNA extraction protocol was provided by the manufacturer. The sample was further analyzed using a quantitative real-time StepOne PCR of the Applied Biosystem, following the protocol of Sánchez-Romero et al. (2009).

### Procedure

A within-subjects repeated measure design was employed, where the subjects completed the three experimental conditions (HIIT, MICT, and CTRL), 1 week apart, following a randomized, counterbalanced order. Before the experimental protocol, 1 week earlier, the subjects visited the laboratory to complete the Pre-participation questionnaires, familiarize themselves with the cognitive test in order to prevent a possible “learning effect,” and provide saliva samples for genetic analysis. On the same day, the subjects also performed cardiorespiratory evaluation, with the data from the latter used to establish exercise intensities for both experimental conditions. Related to cognitive test familiarization, the participants performed a minimum of 5 attempts of each Stroop condition. The learning stabilization was considered when participants made two consecutive attempts with inter-trial variability below 5% in each condition (mean ± SD = 2.86 ± 1.11 between trials). Thus, we respected individual variability in learning the test. Prior studies have also used this percentage of intra-variability as an indication of stable performance in cognitive inhibition tests (Schmit et al., 2015; Pastor et al., 2021). At the same time, participants were also given the order of the experimental sessions as per counterbalancing technique, avoiding the possible influence of session order (Thomas et al., 2015). All visits were scheduled to begin at 9:00 a.m. Caffeine use was restricted, and water consumption was limited to 30 min prior to the visit on the day of saliva sample extraction, following the kit’s manufacturer recommendations.

The experimental conditions (HIIT, MICT, and CTRL) were separated by 1 week. Each one took 20 min to be completed. The cognitive Stroop task and measures of wellbeing and affective state were assessed before and 15 min after the experimental and control conditions, always in the same conditions (same room, table, and chair). Blood lactate was measured before the exercise protocol, 3 min post-exercise, right after the cooldown to estimate the exercise intensity, and 15 min post-exercise was over and cognitive function assessment was about to begin. Subjects in the control condition remained seated while watching a video of general interest. Conversely, both exercise protocols included a 3-min warm-up at 60% MAS, but for the MICT session, participants were required to sustain this running speed until the completion of 20 min of exercise. The HIIT session consisted of 4 bouts of 2 min, with each bout performed at 95% MAS. Two minutes of passive recovery separated high-intensity efforts, while a 3-min cooldown followed the last bout. As with MICT, the entire protocol took 20 min to complete, making the volume of both exercise conditions standardized. The said volume was selected given the previous evidence suggesting that cognitive function, particularly executive control, may see the largest improvements after exercise sessions of similar duration (Chang et al., 2012; Oberste et al., 2019).

### Data analysis

Several sets of analyzes were performed. For all of them, alpha was set at 0.05. Paired *t*-tests and RM ANOVA were used to analyze HR and blood lactate in all three conditions (HIIT, MICT, and CTRL) to ensure adequate manipulation of exercise intensity. For the main analysis of the dependent measures, repeated-measures analysis of variance (ANOVA RM) was calculated to assess differences in both cognitive functions (reaction times and response accuracy in the Stroop test) and psychological wellbeing (subjective vitality and states of positive and negative affect). If the sphericity assumption was not met, Greenhouse–Geisser corrections were applied.

To evaluate the response in the Stroop test, an ANOVA RM 2×3 (Time: Pre-Post × Treatment: HIIT, MICT, and CTRL) were performed for each Stroop Condition. ANOVA RM 2×3 tests (Time × Treatment) were carried out separately for Subjective Vitality, Positive affective state, and Negative affective state.

Additional separate analyzes were conducted for BDNF polymorphism using repeated-measures ANOVA RM with BDNF polymorphism (val/val, val/met or met/met) as a between-subjects factor to establish possible differences in the cognitive response based on the individual’s polymorphism.

Paired *post hoc t*-tests with Bonferroni adjustments for multiple comparisons were performed to identify significant results in ANOVA analysis. As an assessment of effect sizes, partial squared eta (*η*_p_^2^) and Cohen’s d were calculated. The effect sizes are grouped for *η*_p_^2^ as small (≤0.01), medium (≤0.06), and large (≤0.14) and for Cohen’s d as small (≤0.20), medium (≤0.50) and large (≤0.80; Cohen, 1992).

Finally, Pearson’s correlation analyzes (r) were performed to establish possible associations between changes (Δ) (Post value – Pre value) in blood lactate and changes in Stroop Test reaction time and between changes in wellbeing and changes in Stroop Test reaction time. The results were analyzed with JASP 0.16 software (Eric-Jan Wagenmakers, Department of the Psychological Methods University of Amsterdam, Nieuwe Achtergracht 129B, Amsterdam, Netherlands).

## Results

### Exercise intensity parameters

The HR recording revealed higher values for HIIT than MICT both at an absolute level (164.7 vs. 153.1 bpm, *p* < 0.01) and at a relative level (83.4 vs. 77.9%, *p* < 0.01) during the 20-min duration of the session. The average value during high-intensity intervals was 181.5 bpm or 92.3% of the estimated HRmax. Regarding lactate released, there were significant TIME × TREATMENT effect between HIIT, MICT, and CTRL (*p* < 0.01) at both 3 min post-exercise [Δ (mean ± SD): 9.32 ± 4.04 vs. 0.79 ± 1.01 vs. 0.014 ± 0.39 mmol/L, respectively] and 15 min post-exercise [Δ (mean ± SD): 7.31 ± 3.17 vs. 0.41 ± 0.67 vs. 0.019 ± 0.38 mmol/L, respectively] measures.

### Stroop reaction time

Once the adequate manipulation of the Stroop Test at the two time-points of measurement was confirmed (for further details, see “Stroop test check” in the method section), an ANOVA RM 2 (TIME: Pre-Post) × 3 (TREATMENT: HIIT, MICT, CTRL) was carried out to analyze possible differences in the reaction times for each condition of the Stroop test (congruent, neutral and incongruent).

#### Congruent condition

**Figure 1:**
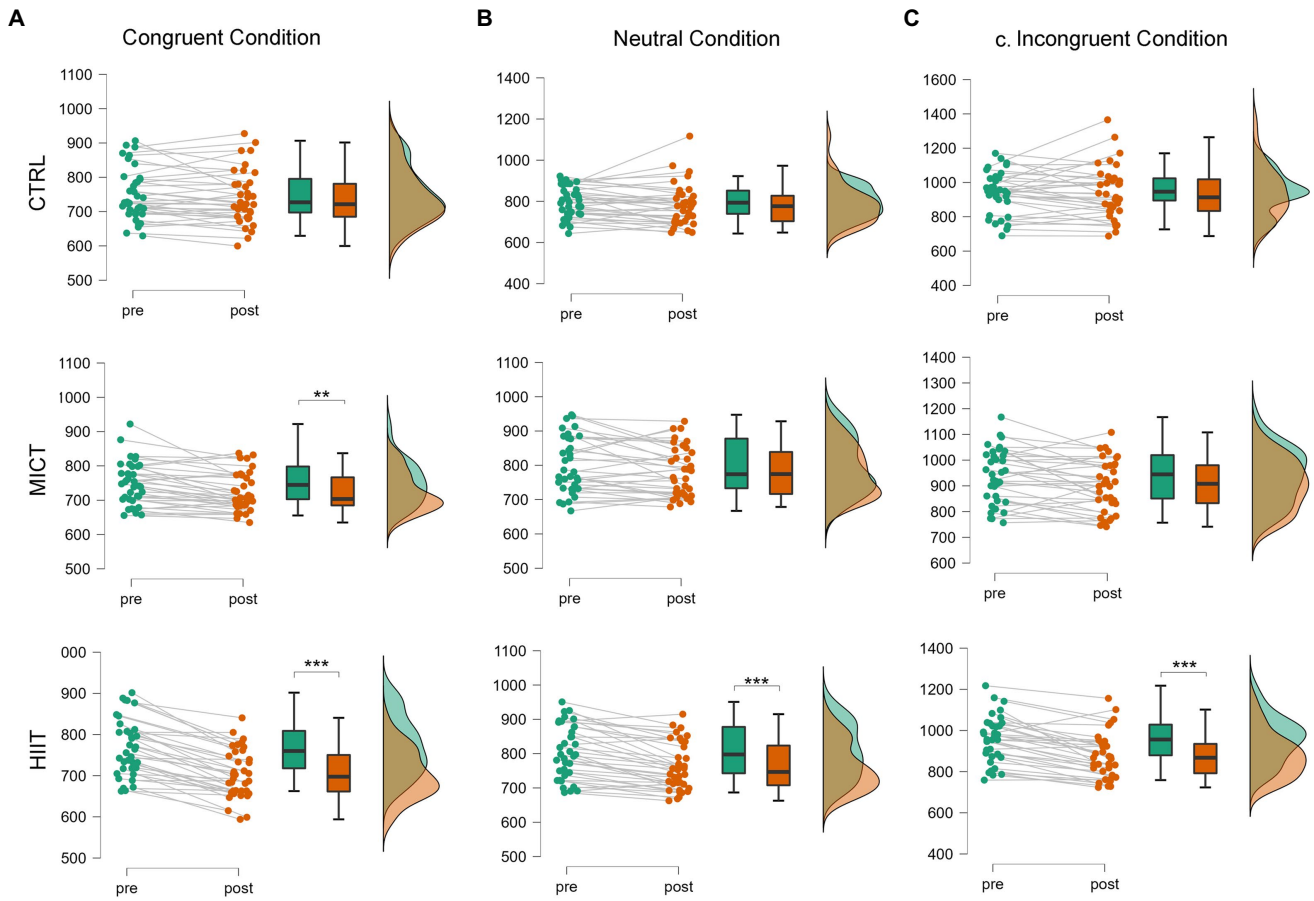
Raincloud plots for time in ms on the three conditions of the Stroop Reaction Time ANOVA RM. Significant effect of time *p < 0.05, **p < 0.01, ***p < 0.001. **(A)** Congruent Condition of the Stroop Test. **(B)** Neutral Condition of the Stroop Test. **(C)** Incongruent Condition of the Stroop Test.

There was a significant main effect of TIME [*F*(1, 35) = 55.69, *p* < 0.001, *η*_p_^2^ = 0.61] while no main effect of TREATMENT (*p* = 0.28) was detected. Regarding TIME × TREATMENT interaction effect for the congruent condition [*F*(2, 70) = 20.14, *p* < 0.001, *η*_p_^2^ = 0.37]. Bonferroni *post hoc* analysis has revealed that both MICT [*t* (35) = 4.10, *p* < 0.01, *d* = 0.68] and HIIT [*t* (35) = 9.46, *p* < 0.001, *d* = 1.58], led to the reduction of the reaction times from the Pre to Post, something that did not occur after the CTRL situation (*p* > 0.05). However, when comparing the Post-session values, we could only find differences between HIIT and CTRL [*t* (35) = 4.35, *p* < 0.001, *d* = 0.73], determined by the effect size of the improvement. Therefore, the effect size of the improvement following the MICT was insufficient to differentiate the Post-response between the MICT session and the CTRL situation (Figure 1A).

#### Neutral condition

There was a significant main effect of TIME [*F*(1, 35) = 16.13, *p* < 0.001, *η*_p_^2^ = 0.31] while no main effect of TREATMENT (*p* = 0.67) was detected. We have also observed a TIME × TREATMENT interaction effect for the neutral condition [*F*(2, 70) = 4.99, *p* < 0.01, *η*_p_^2^ = 0.13]. Bonferroni *post hoc* analysis has demonstrated Pre-Post differences only for the HIIT session [*t* (35) = 5.01, *p* < 0.001, *d* = 0.84] (Figure 1B).

#### Incongruent condition

There was a significant main effect of TIME [*F*(1, 35) = 19.11, *p* < 0.001, *η*_p_^2^ = 0.36] while no main effect of TREATMENT (*p* = 0.15) was detected. Finally, we have also ascertained a TIME × TREATMENT interaction effect for the incongruent condition [*F*(2, 68) = 8.07, *p* < 0.001, *η*_p_^2^ = 0.19]. Bonferroni *post hoc* analysis has revealed a Pre-Post effect only for the HIIT session [*t* (34) = 5.53, *p* < 0.001, *d* = 0.93]. Moreover, after having analyzed Post-session reaction times, we have also observed improvements following HIIT protocol when compared with CTRL [*t* (34) = 3.91, *p* < 0.01, *d* = 0.66] (Figure 1C).

### Wellbeing

#### Subjective vitality

With regards to subjective vitality, a TIME × TREATMENT interaction effect was observed [*F*(1.57, 53.53) = 4.70, *p* = 0.02, *η*_p_^2^ = 0.12] since the opposite trend existed between the exercise protocols (with the tendency to improve) vs. the CTRL situation (with the tendency to get worse). However, the Bonferroni *post hoc* analysis found no statistically significant differences.

**Figure 2:**
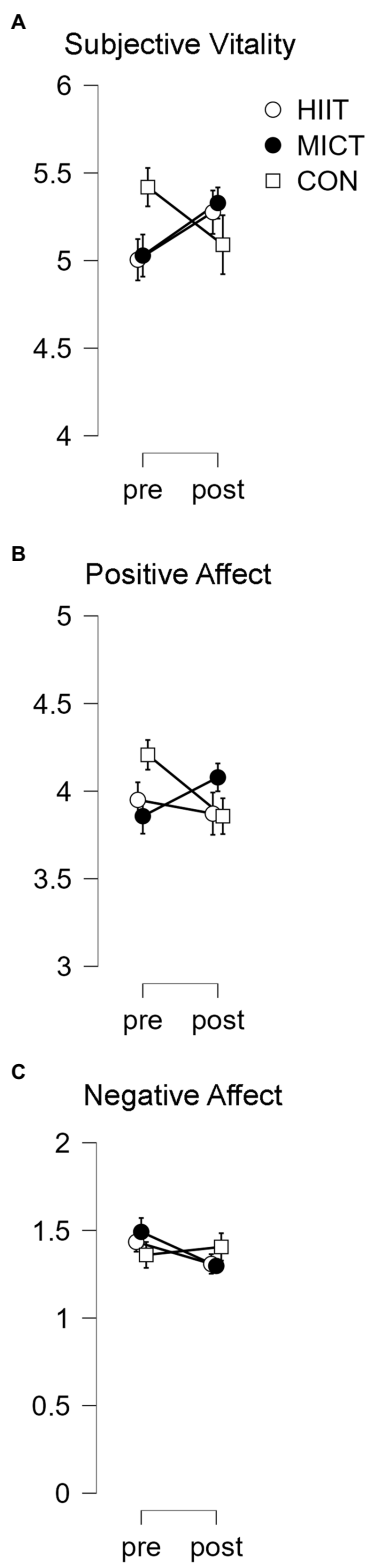
Wellbeing results in pre and post-treatment. Results represent the scale value in the wellbeing questionnaires. **(A)** Subjective vitality results. **(B)** Positive affect results. **(C)** Negative affect results.

Thus, the between-group differences found in ANOVA are the consequence of the distinct evolution of the variables (Figure 2A).

#### Affective state

A TIME × TREATMENT interaction effect was found for the positive Affective state, [*F*(1.57, 53.35) = 4.65, *p* = 0.02, *η*_p_^2^ = 0.12], where the tendency to get worse was true for both HIIT and CTRL, but the opposite trend (the tendency to increase) was revealed for MICT. Nonetheless, the Bonferroni *post hoc* analysis did not find any statistically significant differences. This would suggest that differences observed in the ANOVA RM are concerned with the different trends of the variables and not with the significant changes following interventions (Figure 2B).

Regarding negative affective state, there was no interaction effect between Time and Treatment [*F*(2, 68) = 2.45, *p* = 0.09, *η*_p_^2^ = 0.06] (Figure 2C).

### Interaction of cognitive response with BDNF polymorphism

Genetic analysis has revealed 11 subjects with the val/val coding, 14 with the val/met coding and 11 with the met/met coding.

No between-subject effects were found for any of the included variables based on the BDNF val66met gene factor (*p* > 0.05). The ANOVAs RM 2 × 3 × 3 (TIME × TREATMENT × BDNF val66met) were non-significant in all Stroop conditions: congruent condition [*F*(4, 66) = 0.66, *p* = 0.62, *η*_p_^2^ = 0.04], neutral condition [*F*(4, 66) = 0.47, *p* = 0.75, *η*_p_^2^ = 0.03] nor incongruent condition [*F*(4, 66) = 0.66, *p* = 0.62, *η*_p_^2^ = 0.04].

### Correlation analysis

**Figure 3:**
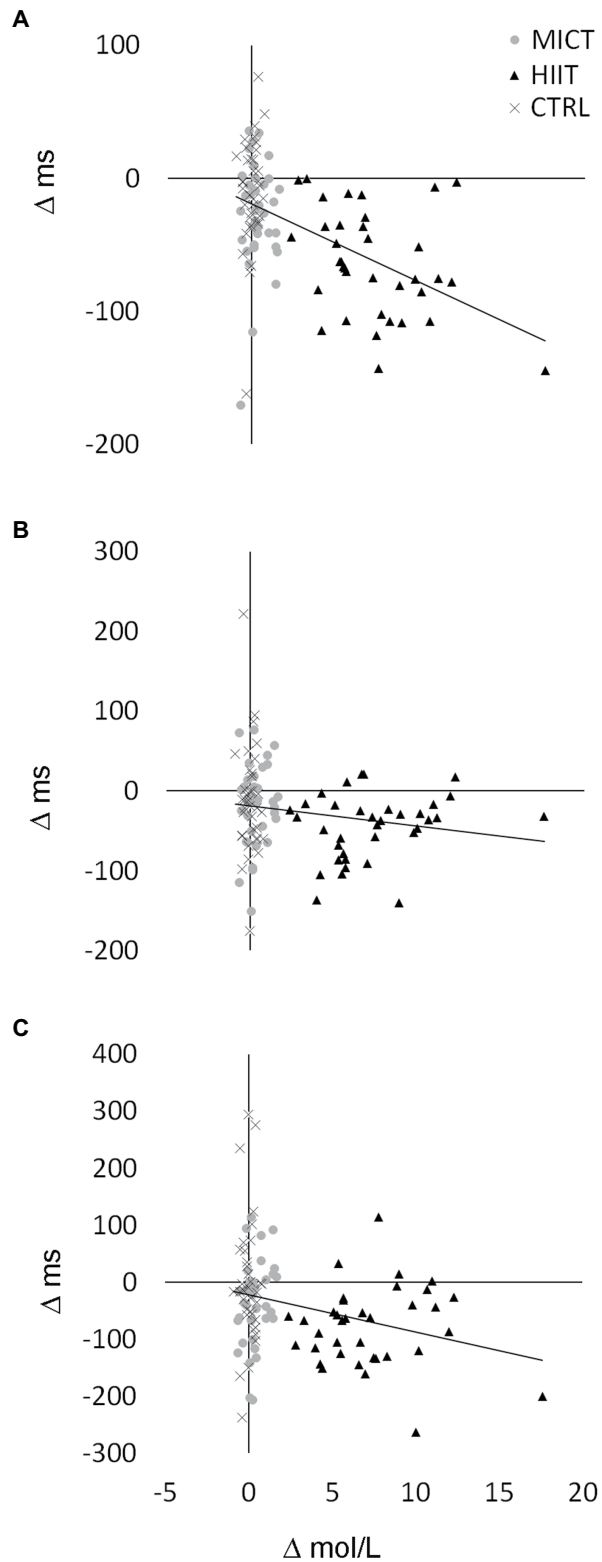
Correlation between lactate increases and improvements in the different conditions of the Stroop test. r and p of the correlations can be shown in text. **(A)** Congruent condition. **(B)** Neutral condition. **(C)** Incongruent condition.

Correlations were identified between changes (Δ) in LA concentration during the cognitive task (at 15 min post-exercise) and Δ in reaction times in congruent condition (*r* = −0.49, *p* < 0.001; Figure 3A), neutral condition (*r* = −0.18, *p* = 0.07; Figure 3B), and incongruent condition (*r* = −0.28, *p* < 0.01; Figure 3C). The analysis of correlations between lactate release at the 15-min post-exercise mark and faster response time in Stroop test conditions separately for each experimental protocol (CTRL, MICT, and HIIT) revealed a correlation between lactate levels and cognitive improvement in congruent test condition following HIIT protocol (*r* = −0.366, *p* = 0.028). This observation did not hold true for any other test condition-protocol pairing (*p* > 0.05 in all of them).

We have also found correlations between changes (Δ) in psychological wellbeing (subjective vitality) and reaction times (Congruent condition; *r* = −0.22, *p* = 0.02), which means the higher is the vitality, the faster is the response.

## Discussion

Our main findings suggest that HIIT elicited superior benefits on processing speed and inhibitory control, both assessed employing the Stroop test. This discovery highlights the crucial role of exercise intensity in producing a cognitive response in young college students. Moreover, we have found a positive association between lactate released from the exercising muscle and cognitive improvements. This association could imply that lactate is one of the mechanisms underlying cognitive gains following acute physical exercise. Regarding wellbeing, a positive trend was observed after both exercise protocols (HIIT and MICT) versus CTRL, without differences between both exercise conditions. Lastly, a genetic marker, BDNF val66met did not determine cognitive response to exercise in young college students.

In the present study, improvements in all three Stroop conditions were only evident after a HIIT session when the cognitive task was administered 15 min after the cessation of exercise. These results agree with the observations of Chang et al. (2012), who showed more pronounced improvements following higher exercise intensities and better results after 15 min. This observation could give us a better understanding of the impact of physical exercise on distinct cognitive domains. On one side, higher performance under congruent and neutral conditions indicates improved basic processing speed (Chang et al., 2019). Nonetheless, we have also observed a positive effect on inhibitory control (i.e., incongruent condition; Chang et al., 2019). In terms of the manipulation of the Stroop task, our data has demonstrated longer reaction times and worse precision index in the incongruent condition than in any of the two remaining stages (congruent and neutral). These latter observations are typically associated with the so-called Stroop effect (Mac Leod, 1991; Milham et al., 2002; Chang et al., 2019). It is also worth noting that we have not found any differences in precision between the experimental protocols (HIIT, MICT, and CTRL). The said finding, combined with the previously mentioned differences between cognitive task conditions, suggests that our manipulation of the Stroop test was adequate, and the positive changes following exercise bout were due to faster responses and not as a result of the compensatory response between speed and precision (Chang et al., 2019).

These results agree with the previously reported effects of exercise on both processing speed and inhibitory control in older (Chu et al., 2015) and young and middle-aged subjects (Chu et al., 2017; Chang et al., 2019). However, traditionally, studies chose to explore the impact of moderate-intensity aerobic exercise and rarely compared this training protocol to distinct modalities (Leahy et al., 2020). There is also a lack of data available on the dose–response relationship and the implications this relationship could have on biological markers and cognitive function (Herold et al., 2019). Nevertheless, there is some evidence in the sample of similar characteristics to ours that between two continuous exercise protocols (moderate and high intensity), moderate-intensity exercise led to an acute improvement of executive function (Loprinzi and Kane, 2015). However, in a more recent work, the same authors have observed an improved memory function after a high-intensity bout, yet again in young college students (Loprinzi et al., 2021). A recent meta-analysis exploring the acute effects of high-intensity exercise (any high-intensity exercise, not limited to HIIT protocols) on executive function has established significant effects of exercise compared to non-exercise control (Moreau and Chou, 2019). This effect was diminished when comparisons were made against other exercising groups, including protocols of light and moderate efforts (Moreau and Chou, 2019). Nonetheless, when focusing on comparisons between HIIT and MICT, some studies have demonstrated that HIIT has been shown to elicit health benefits to a larger extent than MICT. Among these are the effects on cardiovascular and metabolic health (Talanian et al., 2007; Hood et al., 2011) and brain health, where improvements in executive function after HIIT are more pronounced compared to MICT (Tsukamoto et al., 2016; Oberste et al., 2019; Mekari et al., 2020).

In recent years, the growing interest in exploring the mechanisms underlying the cognitive response following physical exercise through the physiological lens brought to attention certain metabolites, including lactate, which has been proposed as a regulator of redox status and neuronal activity (Hashimoto et al., 2021). Recent discoveries point to the relationship between lactate and exercise intensity as a viable explanation of the superiority of HIIT against MICT in triggering a cognitive response (Tsukamoto et al., 2016, 2017; Hashimoto et al., 2018). It is well-known that HIIT generates higher levels of BDNF (Saucedo Marquez et al., 2015), and the latter could be linked to lactate released from the exercising muscle (Ferris et al., 2007). It has been previously reported that lactate could cross the blood–brain barrier (Hashimoto et al., 2018; El Hayek et al., 2019; Nicola and Okun, 2021) and induce BDNF expression in the brain (Müller et al., 2020). In our study, we have observed correlations between lactate release and acute improvement in cognitive response. Interestingly, when we performed a separate analysis for each exercise intensity, we observed the associations between lactate and cognitive improvement in the congruent condition following the HIIT protocol. This improvement in cognitive function, thus, was a consequence of an increased processing speed rather than a better cognitive inhibition. Interestingly, in one of our former studies, we observed how a 10-week High-Intensity Functional Training (HIFT) program induced chronic positive changes in processing speed in young adults (Ballester-Ferrer et al., 2022), similar to the acute effects of exercise in the present study.

In an attempt to associate these acute modifications with the long-term effects of regular practice of physical exercise, it has been reported that baseline BDNF levels were elevated after 3 months of HIFT (Murawska-Cialowicz et al., 2015). These findings make it plausible that chronic changes result from repeated exposure to the acute exercise stimulus and release factors such as BDNF and lactate (Hashimoto et al., 2021). Nonetheless, not all studies have corroborated this relationship. Furthermore, some authors have proposed an inverted U-shaped perspective in the dose–response relationship when reporting the effects of acute exercise (Pontifex et al., 2019). This heterogeneity in the literature could be accounted for by the differences in the selected training modality and exercise protocol, baseline fitness of the participants, the cognitive task employed, or the timing of the cognitive stimuli following exercise (Chang et al., 2012). Concerning exercise protocol, HIIT should not be confused with other types of high-intensity training, some of which did show deleterious effects on cognitive function (Wang et al., 2013; McMorris, 2016). The benefits of interval training (HIIT) are associated precisely with the interval nature of this exercise mode, which aids in lactate liberation, but limits the release of catecholamines and mental stress (Hashimoto et al., 2018). In any case, future research should focus on increasing the evidence through the dose–response relationship perspective in all age groups. Knowledge gained from such studies should help design effective and efficient protocols to improve cognitive function.

Given the putative role of BDNF in cognition, studies exploring the cognitive response to physical exercise should include the Val66Met polymorphism as a moderator variable. The latter could reduce levels of released BDNF and thus, have a direct impact on the cognitive response (Kujach et al., 2019). Along this line of thought, some observational studies have revealed the moderator effect of the Val66Met polymorphism on the exercise-cognition relationship in older adults, along with the benefits associated with the Val/Val SNP (Canivet et al., 2015). However, in our interventional study we could not find significant interactions of the BDNF polymorphism in the exercise-cognition relationship in young college students. This finding agrees with the results reported previously by interventional studies, with the majority revealing the positive effects of exercise on cognition against control conditions, independent of the polymorphism (de Las Heras et al., 2022). Along the same lines, only 31% of interventional studies where the BDNF polymorphism was considered an independent variable reported its moderating effect. Meanwhile, the percentage of observational studies with the same objective was around 82%; however, the results were inconclusive, with the moderating effect of the BDNF polymorphism going in either direction (de Las Heras et al., 2022). The observed differences between the two types of studies could be explained by the presence of distinct mediating factors, such as the participants’ age. For instance, observational studies generally selected older subjects compared to younger samples of interventional research (de Las Heras et al., 2022). It has been previously proposed that Val66Met SNP could determine the reduction in brain reserve during the aging process and define distinct cognitive responses in older populations (Lindenberger et al., 2008). If true, this would suggest that the moderating effect of the BDNF polymorphism in the exercise-cognition relationship becomes apparent later in life, supporting the results of our study on college students. Moreover, aside from age and methodological differences, other moderators such as biological sex, gene interactions, and ethnicity could be influencing the disparate findings (de Las Heras et al., 2022). Given all the above, there does not seem to exist consensus in the literature on the role of the BDNF SNP in the exercise-cognition relationship (de Las Heras et al., 2022), and the benefits of exercise on cognition may well be independent of the latter, as we have observed in our study. Future investigations are required to confirm or refute these assumptions to contribute further to exercise-cognition research.

In our study, along with lactate and BDNF polymorphism as possible mediators of the exercise-cognition relationship, we have also explored the mediating role of behavioral and socioemotional factors (Stillman et al., 2016). Although these factors have shown promising effects on cognition, some variables of this type, such as wellbeing, have been less studied in the literature related to cognition (Stillman et al., 2016). Previous research has revealed how moderate-intensity exercise was conducive to psychological wellbeing; meanwhile, high-intensity exercise could lead to feelings of displeasure (Ekkekakis, 2003). However, some studies have demonstrated a rebound effect, where although at the start of high-intensity effort, the perception of wellbeing was reduced, following a period of approximately 20 min after the exercise cessation, an improvement in wellbeing score was observed (Jung et al., 2014; Malik et al., 2019). In this regard, in our study, psychological variables were assessed some time after the exercise session, and we have observed that psychological variables included in the analysis were modified differently in response to experimental treatments. For instance, a positive trend was observed after both exercise protocols (HIIT and MICT) for subjective vitality; meanwhile, the control situation without exercise revealed a negative trend. In terms of positive affect, negative changes were observed after HIIT and CTRL. However, an increase in positive affect followed MICT. For the negative affect, we could not find any significant changes between experimental conditions. Along the same lines, and in agreement with the results reported by Cervelló et al. (2014), alterations in psychological wellbeing were distinct between experimental conditions, with a positive trend observed after MICT but not after CTRL, where a negative trend was discovered. Concerning HIIT, we could not detect any significant differences between HIIT and MICT in the psychological variables examined. This would imply that exercise intensity had no impact on the psychoemotional state of young college students following exercise. This latter finding somewhat agrees with the previously reported results (Cervelló et al., 2014). Especially when it comes to HIIT, the importance of exercise intensity in influencing psychological state is not clear. For instance, a recent meta-analysis exploring the effects of HIIT on cognitive function and psychological wellbeing has observed a positive trend for both variables after HIIT; however, the effect size was greater for cognitive improvements than for wellbeing (Leahy et al., 2020).

In an attempt to clarify the relationship between cognitive function and psychological wellbeingwe should also point to a negative correlation between changes in processing speed (a congruent condition in the Stroop task) and subjective vitality, suggesting an association exists between improvements in psychological wellbeing and cognitive function (lower reaction times). If true, psychological wellbeing could be a behavioral and socioemotional mediator of cognitive response following exercise (Stillman et al., 2016). We, thus, propose the relevance of evaluating psychological wellbeing in response to exercise, where it could modulate learning and cognitive function (Garcia et al., 2015; Yu et al., 2017). Additionally, from a practical standpoint, taking wellbeing into account could help ensure high adherence rates, especially relevant in clinical populations (Salman et al., 2019). The intrinsic benefit derived from optimal wellbeing is important in and on itself and has placed psychological wellbeing on the list of priorities of the European Union (Miret et al., 2015).

Finally, a few positive aspects of our study should be mentioned. A recent meta-analysis failed to perform a moderator analysis between HIIT and MICT due to the lack of studies comparing the two modalities (Leahy et al., 2020), a limitation that has been addressed in our work. Although some studies have considered younger populations in their samples (Ishihara et al., 2021), in general, recent publications have highlighted the need to conduct rigorous research in understudied groups, including children under 5, adolescents, and young adults (Stillman et al., 2020). To date, a vast majority of published work which attempted to explore the relationship between physical exercise and cognitive function was applied in older adults (Stillman et al., 2020). Nonetheless, according to a recent meta-analysis, most studies exploring the acute effects of high-intensity exercise on executive function included adults aged 19–30 in their samples (Moreau and Chou, 2019).

### Limitations

The present study has some limitations which are worth mentioning. The volume of the exercise sessions in the study was standardized to 20 min based on the previous evidence where this exercise duration was associated with optimal cognitive gains (Chang et al., 2019; Oberste et al., 2019). Conversely, we modified the exercise intensity. Nonetheless, the combination of distinct volumes and intensities could provide insight into the optimum interaction between the two. Our study still revealed superior benefits of HIIT on processing speed and inhibitory control; both evaluated through the Stroop test. These findings have allowed us to infer that HIIT positively impacted executive control (Chang et al., 2019). However, it should be mentioned that executive function is comprised of multiple subdomains, of which some could manifest a unique response to exercise. Thus, future investigations should include different subdomains of executive function in their analysis. At the same time, and guided by previous research (Hashimoto et al., 2018; El Hayek et al., 2019; Nicola and Okun, 2021) we have hypothesized that elevated blood lactate during high-intensity exercise could explain cognitive gains following this exercise mode. In this sense, while we did observe significant associations between lactate concentrations and cognitive improvements, we have only measured blood lactate and inferred that brain lactate levels have also increased in response to exercise. Thus, we could not establish a cause-and-effect relationship and hope that, based on our theoretical assumptions, future studies would attempt to perform additional experiments. We should also highlight the limitation of our sample size concerning the genetic analysis. Given the complexity of ANOVA RM performed (2 × 3 × 3, Time × Treatment SNP), we should have incorporated a sample of 111 individuals to conserve the study power of 0.8, which was out of reach, given our possibilities. Larger samples are required for analysis of similar complexity to ensure the validity of the statistical data.

## Conclusion

Our study demonstrated how exercise could be a helpful tool to improve inhibitory control. Moreover, the effect size of an acute bout of HIIT was larger than that of MICT. As a plausible mechanism underlying these results, lactate released during HIIT was associated with cognitive improvements, ultimately leading us to conclude that exercise above the lactate threshold derives the most benefits for cognition in college students. Moreover, psychological wellbeing, which was positively affected in both experimental conditions (more so in MICT than in HIIT) but not in CTRL, could be related to cognitive gains observed after exercise. As such, our analysis has revealed a link between increased subjective vitality (one of the subdimensions of psychological wellbeing) and improved executive function. These observations highlight the importance of considering multiple mediators of the exercise-cognition relationship.

## Data availability statement

The original contributions presented in the study are included in the article/supplementary material, further inquiries can be directed to the corresponding author.

## Ethics statement

The studies involving human participants were reviewed and approved by the Oficina de Investigación Responsable de la Univerisdad Miguel Hernández de Elche. The patients/participants provided their written informed consent to participate in this study.

## Author contributions

JB-F, BB-L, EC, and DP contributed to conception and design of the study and wrote the sections of the manuscript. JB-F, AR, and BB-L organized the database. JB-F, AR, and DP performed the statistical analysis. JB-F and DP wrote the first draft of the manuscript. All authors contributed to manuscript revision, read, and approved the submitted version.

## Funding

This work was supported by Grant RTI2018-098335-B-I00 funded by MCIN/AEI/10.13039/501100011033/ “ERDF A way of making Europe.”

## Conflict of interest

The authors declare that the research was conducted in the absence of any commercial or financial relationships that could be construed as a potential conflict of interest.

## Publisher’s note

All claims expressed in this article are solely those of the authors and do not necessarily represent those of their affiliated organizations, or those of the publisher, the editors and the reviewers. Any product that may be evaluated in this article, or claim that may be made by its manufacturer, is not guaranteed or endorsed by the publisher.

## References

[ref1] Ballester-Ferrer J. A. Carbonell-Hernández L. Pastor D. Cervelló E. (2022). COVID-19 quarantine impact on wellbeing and cognitive functioning during a 10-week high-intensity functional training program in young university students. Front. Behav. Neurosci. 16:822199. doi: 10.3389/fnbeh.2022.822199, PMID: 35464146 PMC9028760

[ref2] Canivet A. Albinet C. T. André N. Pylouster J. Rodríguez-Ballesteros M. Kitzis A.. (2015). Effects of BDNF polymorphism and physical activity on episodic memory in the elderly: a cross sectional study. Eur. Rev. Aging Phys. Act. 12:15. doi: 10.1186/s11556-015-0159-2 26865879 PMC4748321

[ref3] Cervelló E. Peruyero F. Montero C. Cutre-Coll D. Beltrán-Carrillo V. J. Moreno-Murcia J. A. (2014). Ejercicio, bienestar psicológico, calidad de sueño y motivación situacional en estudiantes de educación física. Cuad. Psicol. Deporte 14, 31–38. doi: 10.4321/S1578-84232014000300004

[ref4] Chang Y. K. Chen F. T. Kuan G. Wei G. X. Chu C. H. Yan J.. (2019). Effects of acute exercise duration on the inhibition aspect of executive function in late middle-aged adults. Front. Aging Neurosci. 11:227. doi: 10.3389/fnagi.2019.00227, PMID: 31551753 PMC6735360

[ref5] Chang Y. K. Chu C. H. Wang C. C. Wang Y. C. Song T. F. Tsai C. L.. (2015). Dose-response relation between exercise duration and cognition. Med. Sci. Sports Exerc. 47, 159–165. doi: 10.1249/mss.0000000000000383, PMID: 24870572

[ref6] Chang Y. K. Labban J. D. Gapin J. I. Etnier J. L. (2012). The effects of acute exercise on cognitive performance: a meta-analysis. Brain Res. 1453, 87–101. doi: 10.1016/j.brainres.2012.02.068 22480735

[ref7] Chu C. H. Chen A. G. Hung T. M. Wang C. C. Chang Y. K. (2015). Exercise and fitness modulate cognitive function in older adults. Psychol. Aging 30, 842–848. doi: 10.1037/pag0000047, PMID: 26652724

[ref8] Chu C. H. Kramer A. F. Song T. F. Wu C. H. Hung T. M. Chang Y. K. (2017). Acute exercise and neurocognitive development in preadolescents and young adults: an ERP study. Neural Plast. 2017, 2631909–2631913. doi: 10.1155/2017/2631909, PMID: 29147585 PMC5632908

[ref9] Cohen J. (1992). Statistical power analysis. Curr. Dir. Psychol. Sci. 1, 98–101. doi: 10.1111/1467-8721.ep10768783

[ref10] Combarros O. Infante J. Llorca J. Berciano J. (2004). Polymorphism at codon 66 of the brain-derived neurotrophic factor gene is not associated with sporadic Alzheimer’s disease. Dement. Geriatr. Cogn. Disord. 18, 55–58. doi: 10.1159/000077736 15084795

[ref11] Cotman C. W. Berchtold N. C. Christie L. A. (2007). Exercise builds brain health: key roles of growth factor cascades and inflammation. Trends Neurosci. 30, 464–472. doi: 10.1016/j.tins.2007.06.011, PMID: 17765329

[ref12] de Las Heras B. Rodrigues L. Cristini J. Weiss M. Prats-Puig A. Roig M. (2022). Does the brain-derived neurotrophic factor Val66Met polymorphism modulate the effects of physical activity and exercise on cognition? Neuroscientist 28, 69–86. doi: 10.1177/1073858420975712, PMID: 33300425

[ref13] Diamond A. (2013). Executive functions. Annu. Rev. Psychol. 64, 135–168. doi: 10.1146/annurev-psych-113011-143750, PMID: 23020641 PMC4084861

[ref14] Egan M. F. Kojima M. Callicott J. H. Goldberg T. E. Kolachana B. S. Bertolino A.. (2003). The BDNF val66met polymorphism affects activity-dependent secretion of BDNF and human memory and hippocampal function. Cells 112, 257–269. doi: 10.1016/s0092-8674(03)00035-7, PMID: 12553913

[ref15] Ekkekakis P. (2003). Pleasure and displeasure from the body: perspectives from exercise. Cogn. Emot. 17, 213–239. doi: 10.1080/02699930302292, PMID: 29715726

[ref16] El Hayek L. Khalifeh M. Zibara V. Abi Assaad R. Emmanuel N. Karnib N.. (2019). Lactate mediates the effects of exercise on learning and memory through SIRT1-dependent activation of hippocampal brain-derived neurotrophic factor (BDNF). J. Neurosci. 39, 2369–2382. doi: 10.1523/jneurosci.1661-18.2019, PMID: 30692222 PMC6435829

[ref17] Ferris L. T. Williams J. S. Shen C. L. (2007). The effect of acute exercise on serum brain-derived neurotrophic factor levels and cognitive function. Med. Sci. Sports Exerc. 39, 728–734. doi: 10.1249/mss.0b013e31802f04c7 17414812

[ref18] Friedman N. P. Miyake A. (2004). The relations among inhibition and interference control functions: a latent-variable analysis. J. Exp. Psychol. Gen. 133, 101–135. doi: 10.1037/0096-3445.133.1.101, PMID: 14979754

[ref19] Garcia D. Jimmefors A. Mousavi F. Adrianson L. Rosenberg P. Archer T. (2015). Self-regulatory mode (locomotion and assessment), well-being (subjective and psychological), and exercise behavior (frequency and intensity) in relation to high school pupils' academic achievement. PeerJ 3:e847. doi: 10.7717/peerj.847, PMID: 25861553 PMC4389278

[ref20] Golden C. (1994). Stroop. Test de Colores y Palabras. Madrid: Tea Ediciones.

[ref21] Hashimoto T. Tsukamoto H. Ando S. Ogoh S. (2021). Effect of exercise on brain health: the potential role of lactate as a Myokine. Meta 11, 1–12. doi: 10.3390/metabo11120813, PMID: 34940571 PMC8709217

[ref22] Hashimoto T. Tsukamoto H. Takenaka S. Olesen N. D. Petersen L. G. Sørensen H.. (2018). Maintained exercise-enhanced brain executive function related to cerebral lactate metabolism in men. FASEB J. 32, 1417–1427. doi: 10.1096/fj.201700381RR, PMID: 29127193

[ref23] Herold F. Müller P. Gronwald T. Müller N. G. (2019). Dose-response matters! – a perspective on the exercise prescription in exercise-cognition research. Front. Psychol. 10:2338. doi: 10.3389/fpsyg.2019.02338, PMID: 31736815 PMC6839278

[ref24] Hood M. S. Little J. P. Tarnopolsky M. A. Myslik F. Gibala M. J. (2011). Low-volume interval training improves muscle oxidative capacity in sedentary adults. Med. Sci. Sports Exerc. 43, 1849–1856. doi: 10.1249/mss.0b013e3182199834, PMID: 21448086

[ref25] Ishihara T. Drollette E. S. Ludyga S. Hillman C. H. Kamijo K. (2021). The effects of acute aerobic exercise on executive function: a systematic review and meta-analysis of individual participant data. Neurosci. Biobehav. Rev. 128, 258–269. doi: 10.1016/j.neubiorev.2021.06.026, PMID: 34147558

[ref26] Jung M. E. Bourne J. E. Little J. P. (2014). Where does HIT fit? An examination of the affective response to high-intensity intervals in comparison to continuous moderate-and continuous vigorous-intensity exercise in the exercise intensity-affect continuum. PLoS One 9:e114541. doi: 10.1371/journal.pone.0114541 25486273 PMC4259348

[ref27] Korhonen J. Linnanmäki K. Aunio P. (2014). Learning difficulties, academic well-being and educational dropout: a person-centred approach. Learn. Individ. Differ. 31, 1–10. doi: 10.1016/j.lindif.2013.12.011

[ref28] Kujach S. Olek R. A. Byun K. Suwabe K. Sitek E. J. Ziemann E.. (2019). Acute sprint interval exercise increases both cognitive functions and peripheral neurotrophic factors in humans: the possible involvement of lactate. Front. Neurosci. 13:1455. doi: 10.3389/fnins.2019.01455, PMID: 32038149 PMC6989590

[ref29] Leahy A. A. Mavilidi M. F. Smith J. J. Hillman C. H. Eather N. Barker D.. (2020). Review of high-intensity interval training for cognitive and mental health in youth. Med. Sci. Sports Exerc. 52, 2224–2234. doi: 10.1249/mss.0000000000002359, PMID: 32301856

[ref30] Lindenberger U. Nagel I. E. Chicherio C. Li S. C. Heekeren H. R. Bäckman L. (2008). Age-related decline in brain resources modulates genetic effects on cognitive functioning. Front. Neurosci. 2, 234–244. doi: 10.3389/neuro.01.039.2008, PMID: 19225597 PMC2622748

[ref31] Loprinzi P. D. Day S. Hendry R. Hoffman S. Love A. Marable S.. (2021). The effects of acute exercise on short-and long-term memory: considerations for the timing of exercise and phases of memory. Eur. J. Psychol. 17, 85–103. doi: 10.5964/ejop.2955, PMID: 33737976 PMC7957845

[ref32] Loprinzi P. D. Kane C. J. (2015). Exercise and cognitive function: a randomized controlled trial examining acute exercise and free-living physical activity and sedentary effects. Mayo Clin. Proc. 90, 450–460. doi: 10.1016/j.mayocp.2014.12.023 25746399

[ref33] Ludyga S. Gerber M. Pühse U. Looser V. N. Kamijo K. (2020). Systematic review and meta-analysis investigating moderators of long-term effects of exercise on cognition in healthy individuals. Nat. Hum. Behav. 4, 603–612. doi: 10.1038/s41562-020-0851-8, PMID: 32231280

[ref34] Mac Leod C. M. (1991). Half a century of research on the stroop effect: an integrative review. Psychol. Bull. 109, 163–203. doi: 10.1037/0033-2909.109.2.163, PMID: 2034749

[ref35] Mackinnon A. J. Jorm A. F. Christensen H. Korten A. E. Jacomb P. A. Rodgers B. J. P.. (1999). A short form of the positive and negative affect schedule: evaluation of factorial validity and invariance across demographic variables in a community sample. Pers. Individ. Differ. 27, 405–416. doi: 10.1016/S0191-8869(98)00251-7

[ref36] Malik A. A. Williams C. A. Weston K. L. Barker A. R. (2019). Perceptual and cardiorespiratory responses to high-intensity interval exercise in adolescents: does work intensity matter? J. Sports Sci. Med. 18, 1–12. PMID: 30787646 PMC6370969

[ref37] Mang C. S. Campbell K. L. Ross C. J. Boyd L. A. (2013). Promoting neuroplasticity for motor rehabilitation after stroke: considering the effects of aerobic exercise and genetic variation on brain-derived neurotrophic factor. Phys. Ther. 93, 1707–1716. doi: 10.2522/ptj.20130053, PMID: 23907078 PMC3870490

[ref38] McMorris T. (2016). Developing the catecholamines hypothesis for the acute exercise-cognition interaction in humans: lessons from animal studies. Physiol. Behav. 165, 291–299. doi: 10.1016/j.physbeh.2016.08.011, PMID: 27526999

[ref39] Mekari S. Earle M. Martins R. Drisdelle S. Killen M. Bouffard-Levasseur V.. (2020). Effect of high intensity interval training compared to continuous training on cognitive performance in young healthy adults: a pilot study. Brain Sci. 10:81. doi: 10.3390/brainsci10020081, PMID: 32033006 PMC7071608

[ref40] Milham M. P. Erickson K. I. Banich M. T. Kramer A. F. Webb A. Wszalek T.. (2002). Attentional control in the aging brain: insights from an fMRI study of the stroop task. Brain Cogn. 49, 277–296. doi: 10.1006/brcg.2001.1501, PMID: 12139955

[ref41] Miret M. Cabello M. Marchena C. Mellor-Marsá B. Caballero F. F. Obradors-Tarragó C.. (2015). The state of the art on European well-being research within the area of mental health. Int. J. Clin. Health Psychol. 15, 171–179. doi: 10.1016/j.ijchp.2015.02.001, PMID: 30487834 PMC6224803

[ref850] Molina J. Castillo I. Pablos C. (2007). Bienestar psicológico y práctica deportiva en universitarios. Motricidad. Eur. J. Hum. Mov. 18, 79–91., PMID: 9768379

[ref42] Moreau D. Chou E. (2019). The acute effect of high-intensity exercise on executive function: a meta-analysis. Perspect. Psychol. Sci. 14, 734–764. doi: 10.1177/1745691619850568, PMID: 31365839

[ref43] Moreau D. Kirk I. J. Waldie K. E. (2017). High-intensity training enhances executive function in children in a randomized, placebo-controlled trial. eLife 6:e25062. doi: 10.7554/eLife.25062, PMID: 28825973 PMC5566451

[ref44] Müller P. Duderstadt Y. Lessmann V. Müller N. G. (2020). Lactate and BDNF: key mediators of exercise induced neuroplasticity? J. Clin. Med. 9:1136. doi: 10.3390/jcm9041136, PMID: 32326586 PMC7230639

[ref45] Murawska-Cialowicz E. Wojna J. Zuwala-Jagiello J. (2015). Crossfit training changes brain-derived neurotrophic factor and irisin levels at rest, after wingate and progressive tests, and improves aerobic capacity and body composition of young physically active men and women. J. Physiol. Pharmacol. 66, 811–821. PMID: 26769830

[ref46] Nicola R. Okun E. (2021). Adult hippocampal neurogenesis: one lactate to rule them all. Neuromolecular Med. 23, 445–448. doi: 10.1007/s12017-021-08658-y, PMID: 33871752

[ref47] Nigg J. T. (2000). On inhibition/disinhibition in developmental psychopathology: views from cognitive and personality psychology and a working inhibition taxonomy. Psychol. Bull. 126, 220–246. doi: 10.1037/0033-2909.126.2.220, PMID: 10748641

[ref48] Oberste M. Javelle F. Sharma S. Joisten N. Walzik D. Bloch W.. (2019). Effects and moderators of acute aerobic exercise on subsequent interference control: a systematic review and meta-analysis. Front. Psychol. 10:2616. doi: 10.3389/fpsyg.2019.02616 31824387 PMC6881262

[ref49] Pastor D. Cervello E. Jimenez M. (2018). “Test Stroop UMH-MEMTRAIN v0.1”, (ed.) C. 580 Valenciana. (Spain: Comunidad Valenciana 09/2018/656). Available at: https://bancodepatentes.gva.es/es/umh/-/asset_publisher/xoxK0ZQPxN2u/content/aplicacion-test-stroop-mem-train

[ref50] Pastor D. Cervelló E. Peruyero F. Biddle S. Montero C. (2021). Acute physical exercise intensity, cognitive inhibition and psychological well-being in adolescent physical education students. Curr. Psychol. 40, 5030–5039. doi: 10.1007/s12144-019-00454-z

[ref51] Pontifex M. B. McGowan A. L. Chandler M. C. Gwizdala K. L. Parks A. C. Fenn K.. (2019). A primer on investigating the after effects of acute bouts of physical activity on cognition. Psychol. Sport Exerc. 40, 1–22. doi: 10.1016/j.psychsport.2018.08.015

[ref52] Rodríguez Barreto L. C. Pineda Roa C. A. Pulido N. d. C. (2016). Propiedades psicométricas del stroop, test de colores y palabras en población colombiana no patológica. Univ. Psychol. 15, 255–272. doi: 10.11144/Javeriana.upsy15-2.ppst

[ref53] Ryan R. M. Frederick C. (1997). On energy, personality, and health: subjective vitality as a dynamic reflection of well-being. J. Pers. 65, 529–565. doi: 10.1111/j.1467-6494.1997.tb00326.x, PMID: 9327588

[ref54] Ryan R. M. Huta V. Deci E. L. (2013). Living Well: A Self-Determination Theory Perspective on Eudaimonia. New York: Springer Science + Business Media.

[ref55] Salman A. Sellami M. Al-Mohannadi A. S. Chun S. (2019). The associations between mental well-being and adherence to physical activity guidelines in patients with cardiovascular disease: results from the Scottish health survey. Int. J. Environ. Res. Public Health 16:3596. doi: 10.3390/ijerph16193596, PMID: 31561424 PMC6801738

[ref56] Sánchez-Romero M. A. Dorado P. Guarino E. Llerena A. (2009). Development of a new genotyping assay for detection of the BDNF Val66Met polymorphism using melting-curve analysis. Pharmacogenomics 10, 989–995. doi: 10.2217/pgs.09.44, PMID: 19530966

[ref57] Saucedo Marquez C. M. Vanaudenaerde B. Troosters T. Wenderoth N. (2015). High-intensity interval training evokes larger serum BDNF levels compared with intense continuous exercise. J. Appl. Physiol. 119, 1363–1373. doi: 10.1152/japplphysiol.00126.2015, PMID: 26472862

[ref58] Schmit C. Davranche K. Easthope C. S. Colson S. S. Brisswalter J. Radel R. (2015). Pushing to the limits: the dynamics of cognitive control during exhausting exercise. Neuropsychologia 68, 71–81. doi: 10.1016/j.neuropsychologia.2015.01.006, PMID: 25576908

[ref59] Stillman C. M. Cohen J. Lehman M. E. Erickson K. I. (2016). Mediators of physical activity on neurocognitive function: a review at multiple levels of analysis. Front. Hum. Neurosci. 10:626. doi: 10.3389/fnhum.2016.00626, PMID: 28018195 PMC5161022

[ref60] Stillman C. M. Esteban-Cornejo I. Brown B. Bender C. M. Erickson K. I. (2020). Effects of exercise on brain and cognition across age groups and health states. Trends Neurosci. 43, 533–543. doi: 10.1016/j.tins.2020.04.010, PMID: 32409017 PMC9068803

[ref61] Stroop J. R. (1935). Studies of interference in serial verbal reactions. J. Exp. Psychol. 18, 643–662. doi: 10.1037/h0054651

[ref62] Talanian J. L. Galloway S. D. Heigenhauser G. J. Bonen A. Spriet L. L. (2007). Two weeks of high-intensity aerobic interval training increases the capacity for fat oxidation during exercise in women. J. Appl. Physiol. 102, 1439–1447. doi: 10.1152/japplphysiol.01098.2006, PMID: 17170203

[ref63] Thomas J. Nelson J. Silverman S. (2015). Research Methods in Physical Activity. Champaign, IL: Human Kinetics Champaign.

[ref64] Titz C. Karbach J. (2014). Working memory and executive functions: effects of training on academic achievement. Psychol. Res. 78, 852–868. doi: 10.1007/s00426-013-0537-1 24389706

[ref65] Tsukamoto H. Suga T. Takenaka S. Tanaka D. Takeuchi T. Hamaoka T.. (2016). Greater impact of acute high-intensity interval exercise on post-exercise executive function compared to moderate-intensity continuous exercise. Physiol. Behav. 155, 224–230. doi: 10.1016/j.physbeh.2015.12.021, PMID: 26723268

[ref66] Tsukamoto H. Takenaka S. Suga T. Tanaka D. Takeuchi T. Hamaoka T.. (2017). Impact of exercise intensity and duration on postexercise executive function. Med. Sci. Sports Exerc. 49, 774–784. doi: 10.1249/mss.0000000000001155, PMID: 27846044

[ref67] Wang C. C. Chu C. H. Chu I. H. Chan K. H. Chang Y. K. (2013). Executive function during acute exercise: the role of exercise intensity. J. Sport Exerc. Psychol. 35, 358–367. doi: 10.1123/jsep.35.4.358 23966446

[ref68] Yu L. Shek D. T. L. Zhu X. (2017). The influence of personal well-being on learning achievement in university students over time: mediating or moderating effects of internal and external university engagement. Front. Psychol. 8:2287. doi: 10.3389/fpsyg.2017.02287, PMID: 29375421 PMC5767243

